# A Rare Case of Cystic Bronchiectasis With Concurrent Pulmonary Aspergillosis in a Rheumatoid Arthritis Patient Receiving Etanercept

**DOI:** 10.7759/cureus.117349

**Published:** 2026-10-02

**Authors:** Hanien Samara, Henrik Ghantarchyan, Twinkle Chandak

**Affiliations:** 1 Internal Medicine, Harbor-UCLA Medical Center, Torrance, USA; 2 Pulmonary and Critical Care Medicine, Internal Medicine, Arrowhead Regional Medical Center, Colton, USA; 3 Pulmonary, Critical Care, Hyperbaric and Sleep Medicine, Loma Linda University Medical Center, Loma Linda, USA

**Keywords:** aspergillosis, case report, etandercept, pulmonology, rheumatoid arthritis

## Abstract

Tumor necrosis factor-alpha (TNF-α) is critical to the body's ability to mount an appropriate inflammatory and immune response to various infections, particularly against intracellular organisms. As such, the immunosuppressive effects associated with TNF-α inhibitors, such as etanercept, may increase susceptibility to opportunistic infections. We report a case of pulmonary *Aspergillus* infection in a 47-year-old female with rheumatoid arthritis receiving etanercept who was found to have underlying cystic bronchiectatic changes. The patient presented with progressive exertional dyspnea and was subsequently found to have right lower lobe cystic bronchiectatic changes with mass-like densities on computed tomography (CT) imaging. Sputum fungal culture was positive for *Aspergillus*, bronchoalveolar lavage (BAL) was positive for *Aspergillus *antigen, and fungal culture from a bronchoscopic biopsy specimen grew *Aspergillus fumigatus*. Other infectious evaluations were negative. The patient was treated with voriconazole, and etanercept was discontinued. This case highlights the diagnostic complexity of interpreting *Aspergillus *isolation in patients with underlying structural lung disease and TNF-α inhibitor exposure.

## Introduction

Tumor necrosis factor-alpha (TNF-α) is a pro-inflammatory cytokine that plays an important role in the pathophysiology of various autoimmune diseases. In addition to mediating inflammatory processes, TNF-α contributes to host immune defense through effects on immune-cell activation and granuloma formation. Within rheumatology, TNF-α inhibitors, including etanercept, have demonstrated clinical benefit in the treatment of autoimmune and inflammatory diseases, such as rheumatoid arthritis and Crohn's disease [1]. Etanercept was the first FDA-approved TNF-α inhibitor for the treatment of rheumatoid arthritis and remains an established therapeutic option, with subcutaneous administration allowing for increased patient convenience [2]. Although infections may occur as a consequence of TNF-α inhibition, *Aspergillus *infection associated with etanercept appears to be uncommon. Compared with other TNF-α inhibitors, such as infliximab and adalimumab, etanercept has been associated with a lower overall risk of serious opportunistic infections [3]. *Aspergillus fumigatus *is the most commonly isolated *Aspergillus *species and is responsible for a substantial proportion of pulmonary *Aspergillus *infections [4]. However, the isolation of *Aspergillus *from respiratory specimens does not by itself establish invasive pulmonary disease, particularly in patients with pre-existing structural lung abnormalities such as bronchiectasis.

Herein, we report a case of pulmonary *Aspergillus *infection in a patient with rheumatoid arthritis receiving etanercept, who was found to have cystic bronchiectatic changes. The case highlights the importance of interpreting *Aspergillus *microbiological findings in conjunction with radiological, bronchoscopic, and clinical findings, particularly in patients with underlying structural lung disease.

This article was previously presented as a meeting abstract at the 2026 American Thoracic Society (ATS) Annual Scientific Meeting on May 17-18, 2026.

## Case presentation

The patient is a 47-year-old female, a never-smoker, with a past medical history including rheumatoid arthritis, anemia of chronic disease, hypertension, and allergic rhinitis, who initially presented to her primary care physician for evaluation of worsening exertional dyspnea of 10 months' duration (shortness of breath with light housework). The patient was treated with leflunomide for approximately five years and was subsequently switched to etanercept two years prior to presentation by her rheumatologist, in view of anemia. The initial chest X-ray ordered by the primary care physician demonstrated a cystic lesion vs. hiatal hernia. It was initially believed that her symptoms were secondary to her large hiatal hernia. She continued to experience worsening shortness of breath for several months afterwards, prompting computerized tomography (CT) of the chest. CT chest (taken two months following initial presentation) revealed right basilar cystic bronchiectatic changes with mass-like densities measuring 1.9 cm and a large left-sided paraesophageal hernia (Figures 1A-1B). She was immediately referred to interventional pulmonology for further workup and evaluation. Initial sputum fungal culture with reflex to rapid identification (FUNID) was positive for *Aspergillus*. Additional infectious labs were negative for HIV and *Coccidioides *antibody. Quantiferon Gold and Xpert MTB/RIF/NAA test (nucleic acid amplification (NAA) test were also negative. Interventional pulmonology proceeded with endobronchial ultrasound (EBUS)-guided bronchoscopic sampling with bronchoalveolar lavage (BAL). BAL was positive for *Aspergillus *antigen. Fungal cultures from biopsy revealed *A. fumigatus*. Acid-fast and Nocardia cultures were negative. *Coccidioides immitis/Coccidioides posadasii*, Histoplasma, *Blastomyces*, and *Cryptococcus *polymerase chain reaction (PCR) tests were all negative. The patient was subsequently started on voriconazole per infectious disease (ID), and etanercept was stopped. Upon outpatient pulmonology follow-up, her pulmonary function tests (PFTs) (Table 1) were notable for severe airway obstruction (Z score: < -4.93) and decreased airway conductance. Lung volumes indicate hyperinflation. Total lung capacity was within normal limits (Z score: -0.85). PFTs were notable for increased airway resistance, but normal diffusion capacity (Figures 2A-2B). Bronchodilator and airway clearance therapy were instituted. The patient has since been educated on the immunosuppressive effects of TNF-α inhibitors and continues to follow up with rheumatology, pulmonology, and infectious disease. 

**Figure 1:**
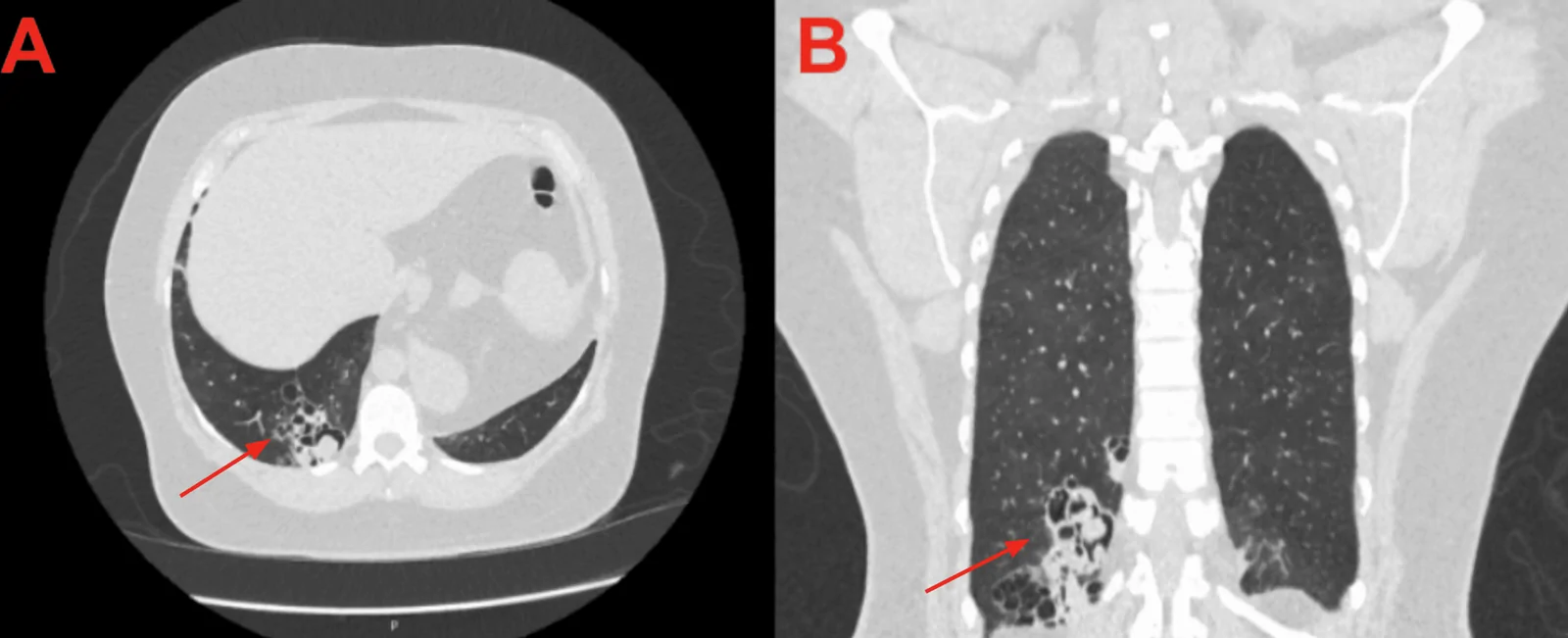
CT chest (transverse (A) and coronal (B) views) showcasing the right lower lobe cystic bronchiectatic changes with mass-like densities and a large left-sided diaphragmatic hernia with an intrathoracic stomach

**Table 1:** Pulmonary function tests with associated flow volume loops (B) indicating severe airway obstruction (Z score: < -4.93), decreased airway conductance and increased airway resistance Total lung capacity (Z score: -0.85) and diffusion capacity are within normal limits. BD: Bronchodilator; FVC: Forced vital capacity; FEV1: Forced expiratory volume in 1 second; FEV1/FVC: Forced vital capacity/forced expiratory colume in one second; FEF 25-75%: Forced expiratory flow at 25-75% of forced vital capacity; ATS: American Thoracic Society; SVC: Slow vital capacity; IC: Inspiratory capacity; ERV: Expiratory reserve volume; TLC: Total lung capacity; Pleth: Plethysmography; FRC: Functional residual capacity; RV: Residual volume; RV/TLC: Residual volume/total lung capacity; Raw: Airway resistance; Gaw: Airway conductance; sRaw: Specific airway resistance; sGaw: Specific airway conductance; LLN: Lower limit of normal; % Predicted: Percent predicted; Sec: Seconds; L: Liters; L/Sec: Liters per second; cmH20: Centimeters of water; 1/cmH20*s: Reciprocal centimeters of water-seconds; AB/AA: Test quality grade

Parameters	Pre-BD				Post-BD			
	Actual	LLN	Z Score	% Predicted	Actual	% Predicted	Volume Change	% Change
Spirometry								
FVC (L)	1.87	2.99	-4.09	49	1.99	52	0.12	+3
FEV1 (L)	0.99	2.39	-4.93	32	1.04	34	0.05	+1
FEV1/FVC (%)	53	70	-3.51	65	53	65		-1
FEF 25-75% (L/sec)	0.38	1.74	-4.34	12	0.42	14		+11
Test Grade (ATS)	AB				AA			
Expiratory Time (Sec)	8.89				9.79			+10
Slow Vital Capacity								
SVC (L)	2.10	2.99	-3.57	55	2.18	57	0.08	+3
IC (L)	1.99		-1.43	72	2.04	74	0.06	+2
ERV (L)	0.11		-3.28	9	0.14	11	0.03	+22
FEV1/SVC (%)	47			58	48	59		+1
Lung Volumes								
TLC (Pleth) (L)	5.05	4.57	-0.85	90				
FRC PL (L)	3.07	2.03	+0.33	106				
RV (Pleth) (L)	2.95	0.89	+2.58	193				
RV/TLC (Pleth) (%)	58	17	+4.55	215				
Airway Resistance								
Raw (cmH20/L/s)	5.88	1.15	+9.40	316				
Gaw (L/s/cmH20)	0.17			24				
sRaw (cmH20*s)	22.92							
sGaw (1/cmH20*s)	0.04	0.14	-4.44	21				

**Figure 2:**
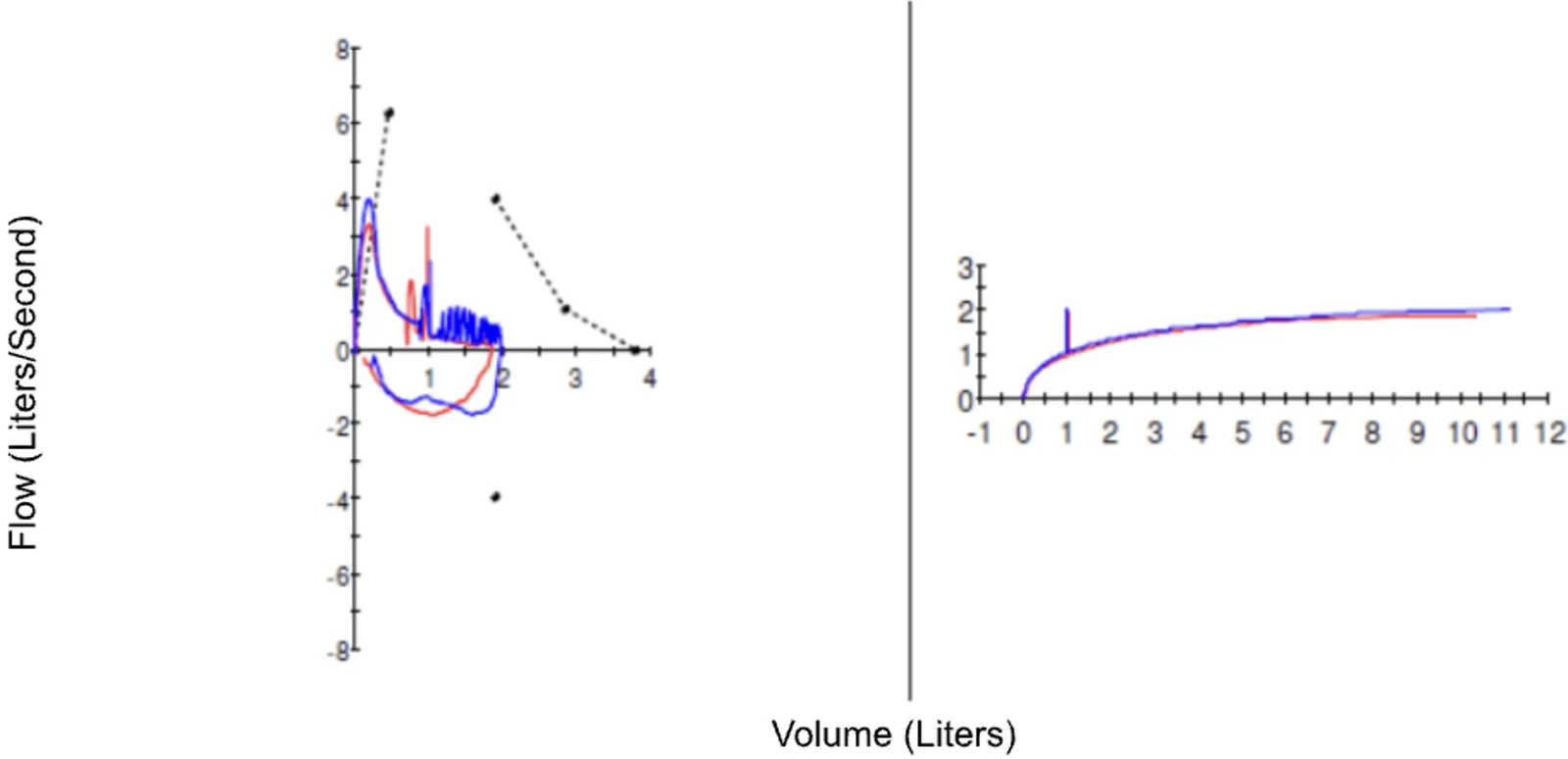
Flow volume loops indicating severe airway obstruction (Z score: < -4.93), decreased airway conductance and increased airway resistance The red line indicates a pre-bronchodilator measurement, whereas the blue line indicates a post-bronchodilator measurement. There is minimal improvement following bronchodilator administration, suggesting no significant bronchodilator response.

## Discussion

This case study provides a unique opportunity to explore the clinical presentation and pathophysiology of *A. fumigatus *infection following treatment with a TNF-α inhibitor (etanercept). A meta-analysis reviewing literature from January 1, 1966, to June 1, 2007 (MEDLINE and PubMed databases) showed 281 documented cases of invasive fungal infections linked with TNF-α inhibitors (infliximab, etanercept, and adalimumab). Of note, only 44 cases (16%) were specifically associated with etanercept [5]. Such literature exemplifies the rarity of invasive fungal infections in the context of TNF-α inhibitor use, with etanercept being the most uncommon.

Immunity in the context of fighting fungal infection relies heavily on CD4 T-cell effector cells and host cell recognition of fungal cell wall elements. As such, neutrophils, macrophages, circulating monocytes, dendritic cells, and natural killer cells are each essential for appropriate immune defense against *A. fumigatus*. Any disruption of these cell lines, such as in the case of TNF-α inhibitor use, could place a patient at risk. Due to TNF-α's role in inflammation and granuloma formation, its suppression has typically led to associated infections with *Mycobacterium tuberculosis*, *Listeria monocytogenes*, and *Histoplasma capsulatum* [6]. Infection with *Aspergillus*, however, is much less common. In the setting of *Aspergillus *infection, pattern recognition receptors (PRRs) signal circulating alveolar macrophages to initiate phagocytosis, with subsequent neutrophil degradation increasing conidial clearance [7]. Inhibiting the inflammatory response triggered by PRR signaling (a known effect of TNF-α inhibitors) is one of the established therapeutic strategies for fighting autoimmune disease; hence, inhibition of TNF-α may therefore alter host defenses against fungal organisms [3].

In this patient, the development of pulmonary *Aspergillus *infection cannot be attributed solely to etanercept therapy. Pre-existing bronchiectatic changes represent an additional potential risk factor, as structural airway abnormalities can predispose patients to persistent microbial colonization and pulmonary infection. The significance of *Aspergillus *isolation must therefore be interpreted carefully in patients with bronchiectasis. *Aspergillus *may be isolated from respiratory specimens because of airway colonization without invasive disease. In this patient, the initial sputum fungal culture was positive for *Aspergillus*; however, subsequent evaluation demonstrated additional evidence of pulmonary *Aspergillus *involvement, including positive *Aspergillus *antigen from BAL and growth of *A. fumigatus *from a bronchoscopic biopsy specimen. These findings were interpreted in conjunction with the patient's radiological abnormalities and clinical presentation rather than relying on the sputum culture alone. Unfortunately, the available case documentation did not include detailed fungal staining results or documented microscopic evidence of fungal hyphal invasion into tissue. Therefore, the positive biopsy culture should not independently be interpreted as definitive proof of tissue-invasive pulmonary aspergillosis. The absence of documented histopathological evidence of invasion represents an important limitation of this case and prevents definitive distinction between invasive pulmonary aspergillosis and other forms of pulmonary *Aspergillus* disease or airway colonization. The clinical differential includes chronic pulmonary *Aspergillus *disease, *Aspergillus *bronchitis, allergic bronchopulmonary aspergillosis, and colonization associated with pre-existing bronchiectasis.

A systematic review investigating case fatality rate (CFR) in the context of invasive aspergillosis (1941 patients pooled from various cohort/case/clinical trial studies published after 1995) displayed an overall CFR of 58%. Patients diagnosed with disseminated aspergillosis experienced an even higher CFR of 88.1%. Notably, the use of amphotericin B deoxycholate and lipid formulations of amphotericin B did not prevent death in 50-75% of patients, further emphasizing the severity of invasive aspergillosis [8]. Although a rare side effect, such data highlight the importance of maintaining high clinical suspicion for invasive aspergillosis in patients on TNF-α inhibitors, in the hopes of preventing death via early treatment and intervention.

The patient's cystic bronchiectatic changes may have occurred in association with her longstanding rheumatoid arthritis, as bronchiectasis has been reported more frequently among patients with rheumatoid arthritis than in the general population [9]. In a large multimodal review of the Korean national health registry, the incidence of bronchiectasis was higher among patients with rheumatoid arthritis than among controls, with a reported incidence of 8.1 versus 3.5 per 1,000 patient-years and a hazard ratio of 2.12 [9]. However, these population-level associations do not establish rheumatoid arthritis as the definitive cause of bronchiectasis in an individual patient. In this case, the underlying bronchiectatic changes should therefore be regarded as a pre-existing structural pulmonary abnormality that may have contributed to susceptibility to pulmonary *Aspergillus *infection. Structural lung disease may be particularly relevant when evaluating *Aspergillus *infection in patients receiving immunomodulatory therapy. A retrospective study from 2004 identified hematologic malignancies and chronic obstructive pulmonary disease among common comorbidities in patients admitted to the intensive care unit with invasive pulmonary aspergillosis [10]. Although these findings cannot be directly extrapolated to this individual patient, they support consideration of underlying pulmonary abnormalities when assessing the risk of pulmonary fungal disease. The patient's severe obstructive ventilatory defect is another notable clinical finding. However, because the PFTs were obtained after the development of her pulmonary symptoms and diagnosis, they cannot establish whether pulmonary function abnormalities preceded her *Aspergillus *infection or increased her risk of developing infection. Further studies would be required to determine whether baseline pulmonary function abnormalities have predictive value for pulmonary *Aspergillus *infection among patients receiving TNF-α inhibitors.

## Conclusions

This case describes pulmonary *Aspergillus *infection in a patient with rheumatoid arthritis receiving etanercept who had underlying cystic bronchiectatic changes. The diagnosis was supported by a combination of radiological, bronchoscopic, and microbiological findings, including positive *Aspergillus *testing from sputum and bronchoalveolar lavage and growth of *A. fumigatus *from a bronchoscopic biopsy specimen. *Aspergillus *isolation in a patient with bronchiectasis does not by itself establish invasive pulmonary aspergillosis, and the absence of documented histopathological evidence of tissue invasion represents an important limitation of this case. TNF-α inhibition may have contributed to susceptibility to *Aspergillus *infection, while pre-existing structural lung disease may have represented an additional risk factor. Overall, further studies are needed to better characterize the relationship between TNF-α inhibitor therapy, structural lung disease, pulmonary function abnormalities, and pulmonary *Aspergillus *infection.

## References

[1] Lee EJ, Song R, Park JN (2010). Chronic necrotizing pulmonary aspergillosis in a patient treated with a tumor necrosis factor-alpha inhibitor. Int J Rheum Dis.

[2] Gerriets V, Goyal A, Khaddour K (2023). Tumor necrosis factor inhibitors. StatPearls.

[3] Yu L, Gao F, Li Y (2024). Role of pattern recognition receptors in the development of MASLD and potential therapeutic applications. Biomed Pharmacother.

[4] Fosses Vuong M, Hollingshead CM, Waymack JR (2026). Aspergillosis. StatPearls.

[5] Tsiodras S, Samonis G, Boumpas DT, Kontoyiannis DP (2008). Fungal infections complicating tumor necrosis factor alpha blockade therapy. Mayo Clin Proc.

[6] Ehlers S (2005). Tumor necrosis factor and its blockade in granulomatous infections: differential modes of action of infliximab and etanercept?. Clin Infect Dis.

[7] Chotirmall SH, Al-Alawi M, Mirkovic B, Lavelle G, Logan PM, Greene CM, McElvaney NG (2013). Aspergillus-associated airway disease, inflammation, and the innate immune response. Biomed Res Int.

[8] Lin SJ, Schranz J, Teutsch SM (2001). Aspergillosis case-fatality rate: systematic review of the literature. Clin Infect Dis.

[9] McDermott GC, Sparks JA (2024). Rheumatoid arthritis and bronchiectasis risk: additional evidence linking autoimmunity and airways disease. Chest.

[10] Meersseman W, Vandecasteele SJ, Wilmer A, Verbeken E, Peetermans WE, Van Wijngaerden E (2004). Invasive aspergillosis in critically ill patients without malignancy. Am J Respir Crit Care Med.

